# Two Laser Treatments Can Improve Tumor Ablation Efficiency of Chemophototherapy

**DOI:** 10.3390/pharmaceutics13122183

**Published:** 2021-12-17

**Authors:** Sanjana Ghosh, Jonathan F. Lovell

**Affiliations:** Department of Biomedical Engineering, University at Buffalo, State University of New York, Buffalo, NY 14260, USA; sghosh22@buffalo.edu

**Keywords:** chemotherapy, doxorubicin, drug delivery, liposomes, phototherapy, photodynamic therapy

## Abstract

Chemophototherapy is an emerging tumor ablation modality that can improve local delivery of chemotherapeutic agents. Long circulating doxorubicin (Dox) in porphyrin-phospholipid (PoP) liposomes (LC-Dox-PoP) has previously been developed as an effective chemophototherapy agent. In the present study, we observed that in mice, LC-Dox-PoP showed enhanced accumulation in human pancreatic tumor xenografts even with suboptimal light doses, as assessed by fluorometric analysis of tissue homogenates and microscopic imaging of Dox and PoP in tumor slices. A second laser treatment, at a time point in which tumors had greater drug accumulation as a result of the first laser treatment, induced potent tumor ablation. Efficacy studies were carried out in two human pancreatic cancer subcutaneous mouse tumor models; MIA PaCa-2 or low-passage patient derived pancreatic cancer xenografts. A single treatment of 3 mg/kg LC-Dox-PoP and an initial 150 J/cm^2^ laser treatment 1 h after drug administration, followed by second laser treatment of 50 J/cm^2^ 8 h after drug administration, was more effective than a single laser treatment of 200 J/cm^2^ at either of those time points. Thus, this study presents proof-of-principle and rationale for using two discrete laser treatments to enhance the efficacy of chemophototherapy.

## 1. Introduction

Chemophototherapy (CPT) is a drug-device combination therapy that merges chemotherapy and phototherapy [1,2,3]. Photodynamic therapy (PDT) is a clinically-used phototherapeutic ablative modality that has been approved for the treatment of several types of dermatological indications and solid tumors [4,5]. Besides destruction of tumor cells, PDT can impact tumor vasculature, leading to vasculature damage and permeabilization, which can enhance tumor delivery of other cargos [6,7,8]. By combining chemotherapy with PDT, enhanced chemotherapy delivery can be achieved, creating an impetus for combining photosensitizers with drug delivery vehicles for CPT. Furthermore, several studies have shown that drugs can be encapsulated inside nanoparticles that can then release the drug upon irradiation with light, leading to improved bioavailability [9,10,11]. Liposomes are self-assembled lipid-vesicles commonly used in the field of drug delivery [12,13]. Liposomal Irinotecan has recently been approved for the treatment of late-stage pancreatic cancer [14,15]. DOXIL is a stable liposomal formulation of doxorubicin (Dox) that was approved by the FDA in 1995 [16].

Liposomal Irinotecan [17,18] and Dox [19,20,21,22] have been reported for CPT. Photoactivatable liposomes can be generated by stably incorporating porphyrin phospholipid (PoP) inside the lipid bilayer. When these photosensitive liposomes are loaded with drugs and irradiated with red light, the entrapped cargo is released, leading to augmented tumoral drug accumulation and better cancer cell killing [23,24,25]. We recently reported a photoactivatable liposomal formulation of Dox, by incorporating small amounts of PoP in its bilayers, that releases Dox when triggered by red light [26]. Long circulating liposomal formulations loaded with chemotherapeutic drugs have shown faster and more efficient tumoral regression as compared to free drugs [27]. Our group has demonstrated a long-circulating liposomal formulation of Dox in liposomes containing 2 mol.% PoP (LC-Dox-PoP) with a circulating half-life of close to 24 h in mice [28] and rats [29].

The choice of the drug-light-interval (DLI) for PDT or CPT is an important consideration. LC-Dox-PoP was shown to be more effective when short DLIs such as 1 h were employed, relative to longer ones such as 24 h [30]. Vascular damage and permeabilization induced at the time of the short DLI enabled subsequent enhanced tumor uptake of the long-circulating chemotherapy drug [31]. In the present study, we aimed to study the impact of a second, later laser treatment on chemophototherapy efficacy once more of both the photosensitizer and the drug has been delivered to the tumor site. To our knowledge, how a second laser treatment could influence and potentially enhance CPT has not yet been reported.

## 2. Materials and Methods

### 2.1. Liposome Preparation and Drug Loading

Lipids were acquired from Corden Pharma International and other materials were acquired from Sigma, if not mentioned otherwise. PoP was synthesized as described [32]. The formulation for LC-Dox-PoP liposomes included 53 mol.% 1,2-distearoyl-sn-glycero-3-phosphocholine (DSPC, Corden Pharma # LP-R4-076 (Boulder, CO, USA)), 40 mol.% cholesterol (PhytoChol, Wilshire Technologies Inc. #57-88-5 (Princeton, NJ, USA)), 2 mol.% PoP and 5 mol.% 1,2-distearoyl-sn-glycero-3-phosphoethanolamine-N-[methoxy(polyethylene glycol)-2000] (MPEG-2000-DSPE, Corden Pharma # LP-R4-039) [28]. To prepare 5 mL of LC-Dox-PoP liposomes, 100 mg of lipids were dissolved in 1 mL ethanol at 60 °C and then 4 mL of 250 mM ammonium sulfate (pH 5.5) was injected into the lipid mixture. The lipid mixture was then extruded 10 times at 60–70 °C through sequentially stacked polycarbonate membranes of 0.2, 0.1 and 0.08 µm pore size in a pressurized nitrogen extruder (Northern Lipids), followed by dialysis (at least twice) in 800 mL solution of 10% sucrose with 10 mM histidine (pH 6.5) to remove ethanol and free ammonium sulfate. Dox (LC Labs # D-4000 (Woburn, MA, USA)) was loaded in the liposomes by incubating Dox in PoP liposomes at 60 °C for 1 h at a Dox: lipid loading molar ratio of 1:5.

### 2.2. Laser Treatment

All murine studies were performed in compliance with the protocols approved by University at Buffalo Institute of Animal Care and Use Committee (IACUC). Following intravenous administration of LC-Dox-PoP at the indicated dosage (typically 3 mg/kg Dox), tumor-bearing mice were anesthetized with isoflurane and tumors were surface-irradiated with 665 nm wavelength light from a fiber-coupled 800 mW RPMC laser diode, LDX-3115-665 (Y2152-1, LDX Optronics Inc. (Maryville, MO, USA)) terminated with a fixed focus collimator. The typical measured laser output was ~100 mW, but was adjusted to achieve a fluence rate of 150 mW/cm^2^, based on the spot area. The typical distance from the tumor to the collimator was ~7 cm and was adjusted to achieve full coverage of the tumor surface area, which had fur removed prior to treatment.

### 2.3. Biodistribution of Dox and PoP

Five to six week old female nude mice (Jackson Laboratories) were inoculated with 5 × 10^6^ MIA PaCa-2 cells on single or both flanks, as indicated. When the sizes of the tumors reached 5–6 mm, mice were intravenously injected with 3 mg/kg LC-Dox-PoP liposomes (Dox basis). One h after drug administration, tumors were irradiated with a laser fluence rate of 150 mW/cm^2^ with a 665 nm laser diode, at the indicated total fluences. Mice were sacrificed at the indicated time points and tissues and blood were collected. To assess the biodistribution of Dox and PoP, tumor and other tissues were homogenized in a Bullet Blender Storm instrument in nuclear lysis buffer (0.25 mol/L sucrose, 5 mmol/L Tris HCl, 1 mmol/L MgSO_4_, 1 mmol/L CaCl_2_, pH 7.6) and homogenates were then extracted overnight with 0.075N HCI, 90% isopropanol at −20 °C. Plasma was collected by centrifuging blood collected in EDTA tubes at 1500× *g* for 15 min and extracted overnight in the same conditions. To analyze the concentration of Dox and PoP in tissue or plasma, the samples were centrifuged at 10,000× *g* for 15 min and supernatants were collected. The amount of drug was determined via fluorescence measurements in a TECAN Safire microplate reader using a standard curve of Dox and PoP using excitation and emission wavelengths of 480 and 590 nm for Dox, and 420 and 670 nm for PoP.

### 2.4. Tumor Growth Inhibition Study

For MIA PaCa-2 tumors, five to six week old female nude mice were inoculated subcutaneously with 5 × 10^6^ MIA Paca-2 cells in the right flank. For the low-passage, pancreatic patient-derived-xenograft (PDX) tumor model (#14312), tumors were implanted in male severe combined immunodeficient (SCID) mice. This model was developed previously at the Roswell Park Comprehensive Cancer [33]. After sacrificing tumor donors under anesthesia, the tumors were immediately harvested and soaked in an ice-cold culture medium and then cut into 2 mm × 2 mm × 2 mm pieces under ice-cold medium. For tumor engraftment, the tumor fragments were quickly implanted subcutaneously into anesthetized mice by making a small incision in the lateral abdominal wall and then closing the incision with a staple. When tumors reached 4–7 mm in diameter, mice were regrouped and treated. The dose of LC-Dox-PoP was 3 mg/kg (Dox basis) unless indicated otherwise. Tumor sizes and mice health were monitored two to three times per week, and tumor volumes were estimated using the ellipsoid formula: Volume = π/6 × L × W^2^, where L and W are the length and width of the tumor, respectively. Mice were sacrificed when tumors exceeded 1.5 cm in size.

### 2.5. Imaging of LC-Dox-PoP Liposomes in Tumor Slices

MIA-PaCa-2 tumor-bearing mice were intravenously injected with LC-Dox-PoP via tail-vein at 15 mg/kg Dox. Mice that received irradiation were treated with a 665 nm laser at a laser fluence rate of 150 mW/cm^2^ and a total fluence of 50 J/cm^2^. Twenty-four h after drug administration, all mice were sacrificed. The tumors were collected and immediately flash-frozen in liquid nitrogen in freezing medium. The tumors were then sliced into ~15 µm thick sections using a Cryostat (H/I Bright OTF5000), placed on glass slides and stored at −20 °C. The tumor slices were imaged using a fluorescence microscope (EVOS FL Auto) to visualize the micro-distribution of Dox and PoP in tumors. Dox was imaged using a GFP filter cube (470 nm excitation; 593 nm emission) and PoP was imaged using a custom filter cube (400 nm excitation; 679 nm emission).

## 3. Results and Discussion

### 3.1. Impact of Suboptimal Light Doses on Anti-Tumor Ablation and Drug Delivery

**Figure 1:**
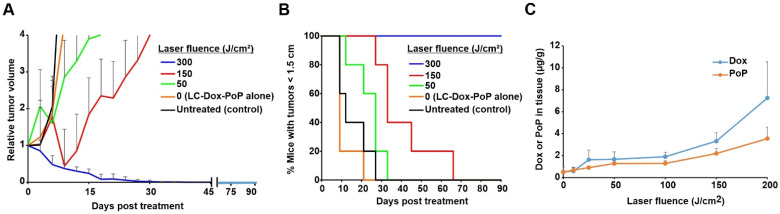
**Chemophototherapy enhances drug delivery even at suboptimal light doses.** Mice bearing MIA PaCa-2 tumors were intravenously injected with 3 mg/kg LC-Dox-PoP liposomes and treated with a 665 nm laser at a 150 mW/cm^2^ fluence rate, at varying laser fluences with a 1 h DLI. (**A**) Relative tumor growth. (**B**) Percentage of mice with tumors less than 1.5 cm. Data show mean ± std. dev. for *n* = 6 mice per group. (**C**) The Dox and PoP concentration in LC-Dox-PoP liposomes in MIA PaCa-2 tumor xenografts laser-treated with indicated fluences (at a fluence rate of 150 mW/cm^2^) with a 1 h DLI. Dox and PoP in tumor were assessed 8 h afar drug administration. Data show mean ± std. dev. for *n* = 4 mice per group.

The anti-tumor efficacy of LC-Dox-PoP liposomes with different laser fluence treatments was assessed. Nude mice bearing MIA PaCa-2 human pancreatic cancer xenografts were untreated or injected intravenously with LC-Dox-PoP liposomes with a Dox dose of 3 mg/kg Dox. Mice were laser-treated with a 665 nm diode laser at a fluence rate of 150 mW/cm^2^ for varying time periods to achieve different total laser fluences of 300, 150, 50 or 0 J/cm^2^ with a 1 h DLI. Figure 1A shows the relative tumor growth of mice that were treated with the same Dox dose but with different laser irradiation parameters. Tumor regression in mice treated with LC-Dox-PoP liposomes with laser irradiation of 300 J/cm^2^ was more efficient than with lesser laser fluences. Figure 1B shows the percentage of mice that maintained tumor sizes less than 1.5 cm. Minimal differences were observed between the groups that were treated with LC-Dox-PoP liposomes with no irradiation and untreated mice. Mice that received laser irradiation with a total fluence of 300 J/cm^2^ showed better tumor inhibition without regrowth. Mice treated with 150 or 50 J/cm^2^ delayed tumor regrowth somewhat, but eventually tumors regrew in all mice from those groups.

To measure the accumulation of Dox in tumors at suboptimal light dosing, female athymic nude mice bearing MIA PaCa-2 human pancreatic tumor xenografts were again injected intravenously with LC-Dox-PoP liposomes with the same drug dose of 3 mg/kg and the tumors were irradiated 1 h later with 665 nm laser light at a fluence rate of 150 mW/cm^2^ at a total fluence of 0, 10, 25, 50, 100, 150 or 200 J/cm^2^. Mice were sacrificed 8 h after drug administration and the tumors were assessed for Dox and PoP distribution (Figure 1C). When tumors were irradiated with 150 J/cm^2^, a non-curative light dose, the amount of Dox and PoP in tumors were found to be 3.3 and 2.2 µg/g, respectively. At 200 J/cm^2^, the amount of Dox and PoP in tumors increased to 7.2 and 3.6 µg/g respectively. On the other hand, without laser treatment, less than 1 µg/g of Dox or PoP was in the tumor. Overall, there was a distinct trend that tumor treatment with increasing laser fluences, even non-curative ones, led to increased delivery of both Dox and PoP. This is consistent with some degree of PDT damage that occurs at the 1 h DLI inducing vascular permeability that enables circulating liposomes to better extravasate into the tumor. Although tumor heating was not measured in this study, a 150 mW/cm^2^, 665 nm laser fluence rate with a LC-Dox-PoP dose of 3 mg/kg was expected to produce minimal heating, as previously a 250 mW/cm^2^, 665 nm laser fluence rate with 7 mg/kg of LC-Dox-PoP did not induce tumor surface heating to greater than 40 °C during 30 min of irradiation [28].

### 3.2. Micro-Distribution of Dox and PoP

**Figure 2:**
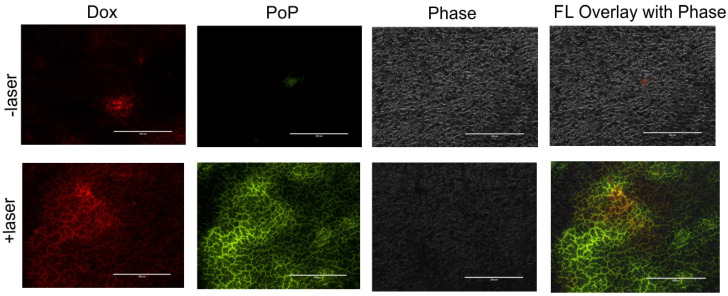
**Micro-distribution of LC-Dox-PoP in tumor slices.** Two groups of mice bearing MIA-PaCa2 tumor xenografts were intravenously administered LC-Dox-PoP (15 mg/kg Dox). 1 h after administration one group of mice was treated using a 665 nm laser at 150 mW/cm^2^ at total laser fluence of 50 J/cm^2^. Mice were sacrificed 24 h after drug administration. Representative tumor slice fluorescent micrographs are shown for *n* = 3 mice per group. Red signal shows Dox distribution and green signal shows PoP distribution. Scale bar, 200 µm.

To assess the micro-distribution of Dox and PoP in tumors, two groups of mice bearing MIA PaCa-2 human pancreatic cancer xenografts were injected with LC-Dox-PoP liposomes at 15 mg/kg Dox. This higher dosage was used to enable visualization of both Dox and PoP in tumor slices with fluorescence microscopy. Mice that received laser treatments were treated using a 665 nm laser at a laser fluence rate of 150 mW/cm^2^ and a total fluence of 50 J/cm^2^. Twenty-four h after drug administration, tumors were collected, flash-frozen in liquid nitrogen and cut into thin slices using a cryostat. As shown in Figure 2, the red signal shows accumulation of Dox and green signal indicates distribution of PoP in the tissues. Tumor slices from mice treated with LC-Dox-PoP with laser irradiation had significantly enhanced accumulation of Dox and PoP compared to the tumor slices from mice that received no laser treatment. The augmented deposition of Dox and PoP in tumors that received laser treatment is consistent with the biodistribution data and demonstrates that laser treatment in CPT induces enhanced drug delivery to irradiated tumors.

### 3.3. Impact of a Second Laser Treatment on Anti-Tumor Efficacy and Biodistribution

**Figure 3:**
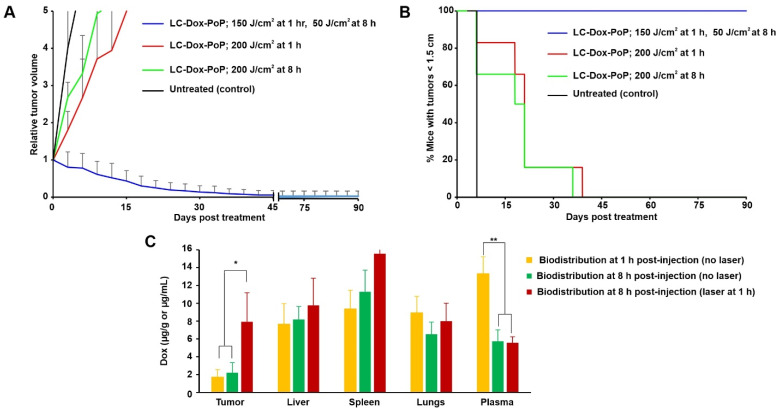
**Impact of a second laser treatment on anti-tumor efficacy and biodistribution.** Mice bearing MIA PaCa-2 tumors were untreated or intravenously injected with 3 mg/kg LC-Dox-PoP liposomes. One group of mice was treated with a 665 nm laser with laser fluence rate of 150 mW/cm^2^ at total fluence of 150 J/cm^2^ at a 1 h DLI and a second laser treatment with 50 J/cm^2^ at a 8 h DLI. The other two groups received laser treatments with a 665 nm laser at a total fluence of 200 J/cm^2^ at either a 1 h DLI or at 8 h DLI respectively. (**A**) Relative tumor growth data. (**B**) Percentage of mice with tumors less than 1.5 cm over time. *n* = 6 mice per group. (**C**) Tissue and plasma biodistribution of Dox and PoP. Mice bearing MIA PaCa-2 tumors were intravenously injected with 3 mg/kg LC-Dox-PoP liposomes. One group was treated with a 665 nm laser with a fluence rate of 150 mW/cm^2^ and a total fluence of 150 J/cm^2^ with a 1 h DLI. Mice were sacrificed at 1 or 8 h, and tumor and other organs were collected for biodistribution analysis. Data show mean ± std. dev. for *n* = 4 mice per group. * *p* < 0.05, ** *p* < 0.01, analyzed by one-way ANOVA with Tukey’s Multiple comparison test in C.

Given that even suboptimal light treatments with a 1 h DLI led to increased LC-Dox-PoP delivery to tumors, we next assessed whether a second laser treatment, at a later time point (when the tumor had elevated drug levels) would be efficient. Mice bearing MIA PaCa-2 human pancreatic cancer xenografts were intravenously injected with LC-Dox-PoP liposomes at a Dox dose of 3 mg/kg. One group of mice was treated with two laser treatments with a 665 nm laser with laser fluence rate of 150 mW/cm^2^. The first treatment was 150 J/cm^2^ with a 1 h DLI and the second treatment was 50 J/cm^2^ with an 8 h DLI. Two other groups of mice were treated with a single laser treatment with the same fluence rate and total fluence of 200 J/cm^2^, with either a 1 h DLI or an 8 h DLI. Figure 3A shows that the tumor growth of mice that received the dual laser treatment decreased over time and shrunk to almost no tumor by day 45. In contrast, mice from the other groups exhibited rapid tumor growth. This indicates the improved efficacy of LC-Dox-PoP liposomes with two discrete laser treatments compared to a treatment with same LC-Dox-PoP dose and total fluence, but with a single irradiation. Figure 3B shows the percentage of mice with tumors smaller than the 1.5 cm endpoint during the study. All mice surpassed this point in the groups that received a single irradiation with a 1 h or 8 h DLI by days 39 and 36, respectively. Mice that were treated with two laser treatments survived through the end of the study.

To account for the enhanced efficacy of the two-treatment approach, the biodistribution of Dox at different time points post drug administration with or without laser treatment was assessed. In the two groups of mice that did not receive laser treatment, one group of mice was sacrificed 1 h after drug administration and the other was sacrificed 8 h after drug administration. Based on the tissue Dox concentration, laser irradiation enhanced drug delivery to the tumor (Figure 3C). The amount of Dox in the tumor of non-irradiated mice 1 h or 8 h after injection was ~1.7 µg/g and ~2.2 mg/g respectively, whereas the amount of Dox in tumors treated with 150 J/cm^2^ (with a 1 h DLI), 8 h after injection was ~8 µg/g. Thus, following an initial laser treatment, several folds more Dox accumulated in the tumor by 8 h. Drug accumulation in other organs was generally similar, although more Dox was found in the plasma 1 h after drug administration compared to 8 h, as expected.

### 3.4. Timing of the Second Laser Treatment

**Figure 4:**
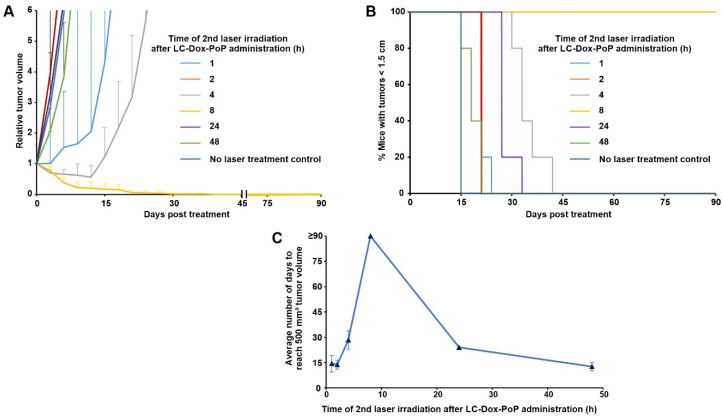
**Timing of a second laser treatment on CPT efficacy.** Mice bearing MIA PaCa-2 xenografts were intravenously injected with 3 mg/kg LC-Dox-PoP liposomes and treated with a 1 h DLI with an initial 665 nm laser treatment of 150 J/cm^2^ at a 150 mW/cm^2^ fluence rate. At the indicated times, a second laser treatment of 50 J/cm^2^ was administered. Mice were sacrificed when the tumors grew to 1.5 cm in size. (**A**) Relative tumor growth. (**B**) Percentage of mice with tumor sizes less than 1.5 cm. (**C**) Average time taken for tumors to grow 500 mm^3^ for mice treated with the indicated timing of the second laser treatment. Data show mean ± std. dev. for *n* = 5 mice per group.

To study the impact of when the second laser treatment is applied, nude mice inoculated with MIA PaCa-2 human pancreatic cancer xenografts were treated with 3 mg/kg LC-Dox-PoP. At the 1 h DLI, mice were treated with a laser fluence of 150 J/cm^2^ using a 665 nm laser at a laser fluence rate of 150 mW/cm^2^. Mice received a second laser treatment in the same conditions, but with a total fluence of 50 J/cm^2^ at different time points: 1 h (immediately following the initial treatment), 2 h, 4 h, 8 h, 24 h or 48 h. Tumor regression was significantly enhanced when the second laser treatment occurred at 8 h, followed by the treatment at 4 h and 24 h respectively (Figure 4A). Mice receiving the second laser treatment at 8 h survived for the longest period of time in the study (Figure 4B). Mice receiving the second laser treatment at a 4 h or 24 h DLI survived up to 42 days and 33 days, respectively. Figure 4C shows a comparative analysis of the average time, in days, taken for the tumors to grow to a volume > 500 mm^3^. The amount of time taken to reach 500 mm^3^ was highest when the mice received their second laser treatment at the 8 h DLI (these mice survived the entire study duration of 90 days), followed by the second laser treatment at 4 h (27 days), 24 h (24 days),1 h (12 days), 48 h (12 days), 0 h (11 days) and no laser treatment (9 days). This indicates the temporal significance of the second laser treatment. Even though all tumor-bearing mice were irradiated with the same total fluence, strong efficacy only occurred when the second laser treatment was optimally timed. It is known that the therapeutic outcome of CPT is affected by DLI as it determines the tumoral deposition of a liposomal drug at a particular time after the administration of liposomes. The first laser dose is assumed to increase permeability of tumor vasculature by PDT-mediated damage of tumor blood vessels and the hyperthermia-induced vascular permeability effect. The damaged tumor vessels allow better accumulation of the liposomal drug. The second laser dose induces further PDT damage and possibly light-triggered drug release from LC-Dox-PoP deposited in the tumors as a result of the first laser treatment. This combined effect of two laser doses allows for an enhanced therapeutic effect when the second laser dose is administered at 8 h DLI. We previously observed that 8 h is a time point in which a high amount of drug is delivered to the tumor following CPT [31]. Therefore, it is likely that irradiating the tumor for the second time when the intratumoral concentration of LC-Dox-PoP is close to its highest level gives rise to improved PDT and drug release effects.

### 3.5. Impact of Second Laser Treatment on PDX Tumor Inhibition

**Figure 5:**
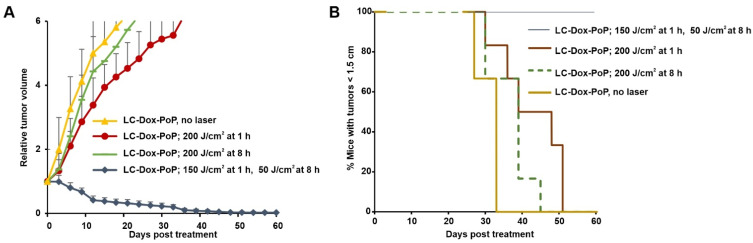
**Impact of a second laser treatment for pancreatic PDX tumors.** SCID mice bearing low-passage human pancreatic PDX tumors were treated with 3 mg/kg LC-Dox-PoP liposomes and treated with a 665 nm laser with a first treatment of 150 J/cm^2^ at a 1 h·DLI and second laser treatment with 50 J/cm^2^, or alternatively with a laser single treatment of 200 J/cm^2^ at a 1 h or 8 h·DLI. (**A**) Relative tumor growth. Data show mean ± std. dev. for *n* = 6 mice per group. (**B**) Percentage of mice with tumors < 1.5 cm over time.

To determine the therapeutic efficacy of dual laser treatments in a second tumor model, low-passage pancreatic patient-derived tumor xenografts (PDX) in SCID mice were used. When tumors reached 5–7 mm in diameter, mice were injected with LC-Dox-PoP liposomes (3 mg/kg·Dox) and then treated with a 665 nm laser with a laser fluence rate of 150 mW/cm^2^ and a fluence of 150 J/cm^2^ at 1 h·DLI and 50 J/cm^2^ at 8 h·DLI, or with a single tumor irradiation of 200 J/cm^2^ at a 1 or 8 h DLI. Figure 5A shows the relative tumor growth of mice. Figure 5B shows the percentage of mice with tumors less than 1.5 cm. By day 12, the tumors of mice that received two discrete laser treatment were reduced to almost half their size, whereas the tumors of mice from other groups had increased their volume by threefold. Mice that received a second laser treatment showed progressive regression in tumor volume over time, gradually shrinking to almost no tumor by day 40. On the other hand, at this time point, several mice from the other groups had developed tumors greater than 1.5 cm. This shows that the second laser treatment is more efficient in shrinking tumors as compared to a single treatment at 1 h or 8 h DLI. This further establishes the premise that a second laser treatment can control tumor growth in CPT.

## 4. Conclusions

Herein, we demonstrated that CPT can be enhanced by applying two laser treatments to the tumor. Even at the same total cumulative fluence, two laser treatments were shown to be more effective than one in mice bearing subcutaneous MIA PaCa-2 or PDX human pancreatic tumors. Although single treatment CPT can be highly efficient, in cases where there are large tumors or it is difficult to apply sufficient light to treat the entire tumor volume, this approach could be of benefit. Tumor growth inhibition studies demonstrated that LC-Dox-PoP was highly effective when two laser treatments were applied at 1 h (to trigger drug deposition) and 8 h (to irradiate the tumor containing a high concentration of Dox and PoP). This timing appeared to be important, since efficacy diminished when the second laser treatment was at other time points. Enhanced deposition of LC-Dox-PoP due to vascular permeabilization of tumors from the first laser treatment followed by tumoral destruction by PDT and drug release from the second laser treatment results in a potent CPT ablative strategy.

## Data Availability

Data are available on reasonable request.
